# An Efficient Coded Streaming Using Clients’ Cache

**DOI:** 10.3390/s20216220

**Published:** 2020-10-31

**Authors:** Tae-Won Ban, Woongsup Lee, Jongyeol Ryu

**Affiliations:** Department of Information and Communication Engineering, Gyeongsang National University, Tongyeong 53064, Korea; twban35@gnu.ac.kr (T.-W.B.); wslee@gnu.ac.kr (W.L.)

**Keywords:** edge caching, streaming, multimedia, coded streaming, steady-state probability

## Abstract

As multimedia traffic has been increasing and is expected to grow more sharply, various technologies using caches have been attracting lots of attention. As one breakthrough technology to deal with the explosively growing traffic, exclusive OR (XOR)-based index coding has been widely investigated because it can greatly enhance the efficiency of network resource by reducing the number of transmissions. In this paper, we investigate how to apply XOR-based index coding to large-scaled practical streaming systems for video traffic that accounts for more than 70% of total Internet traffic. Contrary to most previous studies that have focused on theoretical analysis of optimal performance or development of optimal index coding schemes, our study proposes a new XOR coding-based video streaming (XC). We also propose a new grouping algorithm for creating XC groups while guaranteeing the complete backward compatibility of XC with existing streaming schemes such as unicast (UC), multicast (MC), and broadcast (BC). The performance of the proposed scheme is analyzed in two steps. First, the behavior of video contents in caches at clients is modeled as a Markov chain, and the steady-state probabilities and caching probabilities for each piece of video content are derived. Based on the probabilities, the performance of the proposed system is then analyzed in terms of the average number of connections that each client requires in order to receive one video content. Our numerical results show that the proposed video streaming scheme using XC can reduce the average number of transmissions by up to 18%, compared to the conventional scheme.

## 1. Introduction

A recent study showed that the total amount of Internet traffic will increase three-fold over five years from 2017 to 2022, and video traffic would be growing more sharply than other types of traffic [1]. More specifically, it was predicted that the video traffic that accounted for about 75% of total Internet traffic in 2017 would increase to about 82% in 2022. It was also revealed by another interesting study that the most popular 50 videos accounted for almost 80% of the total number of views in a major over-the-top (OTT) service provider, YouTube [2]. Video service providers deploy huge content delivery systems and manage their own systems to meet the increasing demand, but the approach causes a tremendous cost to the companies [3]. An alternative is to use caching [4,5]. If a requested content exists in the cache of a nearby proxy server, clients can receive the cached copy, which typically reduces delivery time. Recently, the concept of caching has been extended to wireless multimedia streaming systems to cope with the sharply increasing data traffic by offloading increasing video traffic or improving the spectral efficiency of wireless networks [6,7,8,9,10,11,12,13,14,15]. The problem of caching placement was investigated, and some state-of-the-art solutions were introduced in [6,7,8,9]. A dynamic deep reinforcement learning-based management framework for virtual cache slicing was proposed to manage the limited cache resources when service providers share a common physical infrastructure [6]. A probabilistic caching placement was investigated to control cache-based channel selection diversity and network interference in a stochastic wireless caching helper network [7]. A tradeoff between the content diversity gain and the cooperative gain according to content placements was investigated and a probabilistic content placement to optimally balance the tradeoff was proposed [8]. Device-to-device (D2D) caching network, where each node caches some contents and another node can receive these contents directly from its nearby nodes instead of a remote service provider, was investigated in [10,11].

Contrary to previous studies that considered transmitters with cache and studied how to use the transmitters’ cache efficiently, some studies focused on clients with cache [12,13,14,15,16,17,18,19]. If a client wants to play a content which has been stored in its cache, the content can be played directly from the cache with no outbound connection. A joint problem of content pushing and recommendation for cache-enabled mobile users was formulated and a reinforcement learning-based framework for maximizing the profit of mobile network operators was proposed in [12]. Novel transmission techniques based on state-of-the-art interference management schemes such as interference cancellation, zero-forcing, and interference alignment were proposed for cache-aided radio access networks where edge nodes and user equipment are both equipped with cache [13]. An erasure broadcast network was investigated considering two disjoint sets of receivers: a set of weak receivers with an equal size of cache and a set of strong receivers with no cache [14]. It was proposed to exploit limited cached packets as side information to cancel incoming interference at the receiver side in a stochastic network where the random locations of base stations and users are modeled by Poisson point process [15].

Moreover, more advanced transmission schemes that use the contents stored in clients’ cache more efficiently were proposed to improve the efficiency of limited network resource [16,17,18,19]. If a transmitter has the perfect information of contents stored in clients’ caches, the transmitter can transmit multiple contents requested by different clients at once by using XOR index coding and each client can recover its content by performing XOR operations of the XOR coded data and contents stored in its cache, which can enhance the efficiency of network resource by reducing the number of transmissions [16]. The exact broadcast rate which had remained unknown was analyzed using linear programs [17]. A simple inner bound was established on the general index coding problem and it was demonstrated that the inner bound is tight for all index coding problems of up to five messages [18]. A pruning algorithm that can find the optimal clique cover index code and transmission time allocation for minimizing wireless outage probability was proposed to reduce a computational complexity of a brute-force searching algorithm [19]. Most of aforementioned studies were devoted to theoretical research to analyze optimal performance or to develop optimal coding schemes, and they are not thus sufficient to be applied to real large-scaled video streaming systems. In addition, the real advantages of the index coding-based video streaming and its compatibility with existing streaming schemes have never been verified.

In this paper, we thus propose a new video streaming system that supports XC. We also propose a new grouping algorithm for creating XC groups while guaranteeing the complete backward compatibility of XC with existing streaming schemes such as UC, MC, and BC. The performance of the proposed system is analyzed and compared with that of a conventional streaming system to verify its gain. We first model the behavior of contents in caches at clients as a Markov chain and derive the steady-state probabilities and caching probabilities for each video to mathematically analyze the statistical characteristics of caches at clients in video streaming systems. Based on the statistical characteristics of caches, the performance of the proposed system is analyzed in terms of the average number of connections that each client requires in order to receive one video content. The proposed scheme can be widely applied for various types of networks including mobile networks and sensor networks with video streaming.

The rest of this paper is organized as follows. In Section 2, a video streaming system where clients are equipped with cache, and we derive the steady-state probabilities and caching probabilities for each video based on a Markov chain. A new video streaming system for supporting XC is proposed and a grouping algorithm for the proposed streaming system is also described in Section 3. Numerical results are shown in Section 4, and the conclusions of this paper are drawn in Section 5.

## 2. A Streaming System Using Clients’ Cache

**Figure 1:**
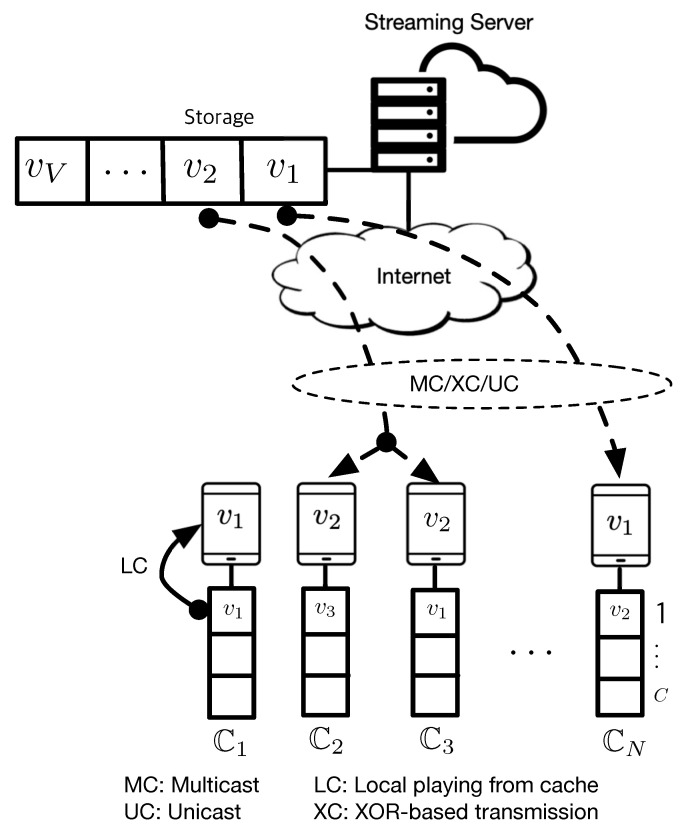
A video streaming system where each client is equipped with a cache size *C*.

We investigate a video streaming system with a single streaming server and *N* clients, which is illustrated in Figure 1. The streaming server and each client are all equipped with cache that can store up to *V* and *C* videos (V>C), respectively. It is assumed that all videos have the same size. $C_{n}$ denotes the cache of client *n* and $|C_{n}|=C\;\forall \;n.$ If each client plays video contents independently of others, the statistical characteristics of cache at each client will be identical and hence, we omit the index *n* for a simplicity of the notation, now and hereafter. C(s) denotes the video that is stored in the *s*-th memory of a cache C, where 1≤s≤C. Thus, C(1) and C(C) indicate the most recently used video and the least recently used video, respectively. $L_{s}$ denotes a set of videos used later than C(s) and is given by

(1) $$L_{s}=\left\{\begin{array}{cc} \{C(1),\ldots ,C(s-1)\}, & \mathrm{if}\;s\neq 1\\ \emptyset , & \mathrm{if}\;s=1. \end{array}\right.$$

$R_{s}$ denotes a set of videos used earlier than C(s) and is given by

(2) $$R_{s}=\left\{\begin{array}{cc} \{C(s+1),\ldots ,C(C)\}, & \mathrm{if}\;s\neq C\\ \emptyset , & \mathrm{if}\;s=C, \end{array}\right.$$

and can be also obtained by $R_{s}=C\backslash L_{s}\backslash \left\{C\left(s\right)\right\}=C\backslash \left\{C\left(1\right),\ldots ,C\left(s\right)\right\}$.

The normalized popularity of a video that ranks *v*-th can be predicted by Zipf distribution [20,21], which is given by

(3) $$f_{v}=\frac{1/v^{\alpha }}{\sum_{n=1}^{V}\left(1/n^{\alpha }\right)},\;1\leq v\leq V,$$

where $f_{v}$ satisfies $\sum_{v=1}^{V}f_{v}=1$ and α is the exponent characterizing Zipf distribution [22]. Zipf distribution with α=0 turns into a uniform distribution, by which all *V* videos have the same normalized popularity, $\frac{1}{V}$. We use the least recently used (LRU) algorithm as a cache replacement strategy [23], by which clients’ caches are autonomously updated without consuming extra network resource after streaming a video content. If a client wants to play a video content *v* that is not stored in the cache, which denotes v∉C, the content *v* will be streamed by one of MC, XC, or UC. After playing the content *v*, LRU discards C(C), which is the least recently used one, and the content *v* will be stored in the first place of the cache after shifting all contents in the cache to the right. Thus, C will be updated as C={v,C(1),…,C(C−1)}. On the other hand, if a client wants to play a content *v* stored in the *s*-th place of the cache, which denotes v=C(s),1≤s≤C, the content *v* will be directly played by local playing (LC) from the cache without using any network resource for streaming. Then, the content *v* will be re-located to the first place of the cache to ensure that the most recently used content will stay in the cache the longest. The contents in $L_{s}$ will be only shifted to the right, while there will be no change on contents in $R_{s}$. Thus, C will be updated as C={v,C(1),…,C(s−1),C(s+1),…,C(C)}. The case of s=1 denotes that the same content has been being played twice in a row and there is then no change on the cache.

**Figure 2:**
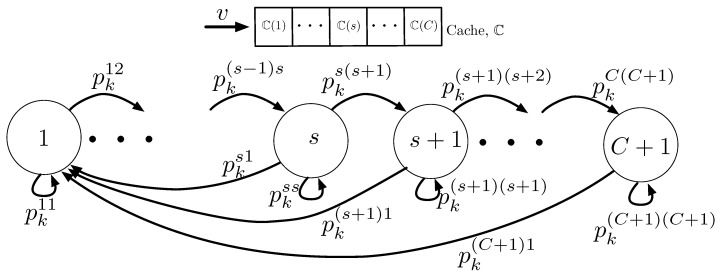
A state transition diagram of a video *k* in a cache with a length of *C*.

A certain content k(1≤k≤V) might be stored in the client’s cache or might not be stored. In the case that content *k* is stored in the cache, the content *k* can have *C* states based on its stored location. Thus, each content *k* can have total (C+1) states in C. The (C+1)-th state denotes that the content is not stored in the cache. The behavior of content *k* in the cache can be modeled by a Markov chain, as depicted in Figure 2. Transition probability $p_{k}^{sj}$ denotes a probability that a content *k* staying at state *s* moves to state *j* after a client plays the content *v*.

For the case of s=1, a content *k* staying in the first place has two possibilities of transition. If v=k, which means that a client plays the content *k* by using LC, the video *k* will be still staying in the first place with no transition. Otherwise, it will move to the second place. Two transition probabilities are thus given as

(4) $$\begin{array}{ccc} p_{k}^{11} & = & \mathrm{Pr}\left[v=k\right]=f_{k} \end{array}$$

(5) $$\begin{array}{ccc} p_{k}^{12} & = & \mathrm{Pr}\left[v\neq k\right]=1-p_{k}^{11}=1-f_{k}. \end{array}$$

For the case of 2≤s≤C, a content *k* has three possibilities of transition. If v=k, then the content *k* will be played by LC and move to the first place after being played. If $v\in L_{s}$, the content *k* will have no transition. Finally, if $v\in R_{s}\;\mathrm{or}\;v\notin C$, the content *k* will be shifted to the right and thus move to the (s+1)-th place. The corresponding transition probabilities are given as

(6) $$\begin{array}{cc} p_{k}^{s1} & =\mathrm{Pr}\left[v=k\right]=f_{k} \end{array}$$

(7) $$\begin{array}{cc} p_{k}^{ss} & =\mathrm{Pr}[v\in L_{s}] \end{array}$$

(8) $$\begin{array}{cc} \; & =\sum_{X\in {}_{{\tilde{V}}_{k}}\;P_{s-1}}\frac{\prod_{x\in X}f_{x}}{\sum_{X\in {}_{{\tilde{V}}_{k}}\;P_{s-1}}\prod_{x\in X}f_{x}}\sum_{x\in X}f_{x} \end{array}$$

(9) $$\begin{array}{cc} p_{k}^{s(s+1)} & =\mathrm{Pr}\left[v\in R_{s}\;\mathrm{or}\;v\notin C\right]=1-p_{k}^{s1}-p_{k}^{ss}, \end{array}$$

where ${\tilde{V}}_{k}$ denoting the index set of contents except for *k* can be obtained by

(10) $${\tilde{V}}_{k}=V\backslash \left\{k\right\}=\left\{1,2,\ldots ,k-1,k+1,\ldots ,V\right\},$$

and ${}_{X}\;P_{n}$ denotes a set that consists of all possible permutations of choosing *n* objects from a set X. Thus, ${}_{{\tilde{V}}_{k}}\;P_{s-1}$ denoting a set of all possible $\frac{(V-1)!}{(V-s)!}$ permutations of (s−1) contents that can be chosen from the set ${\tilde{V}}_{k}$ represents all possible combinations of $L_{s}$. For the case of s=C+1, the content *k* has two cases of transition. If v=k, the content *k* move to the first place after being played. Otherwise, the content *k* will be discarded from the cache. The corresponding transition probabilities are given as

(11) $$\begin{array}{ccc} \;\;p_{k}^{(C+1)1}\;\; & \;\;=\;\; & \;\;\mathrm{Pr}\left[v=k\right]=f_{k} \end{array}$$

(12) $$\begin{array}{ccc} \;\;p_{k}^{\left(C+1)(C+1\right)}\;\; & \;\;=\;\; & \;\;\mathrm{Pr}\left[v\neq k\right]=1-p_{k}^{(C+1)1}=1-f_{k}. \end{array}$$

A square matrix consisting of state transition probabilities for the content *k* is defined as

(13) $$P_{k}≜\left[p_{k}^{ij}\right],\;1\leq i\leq C+1,\;1\leq j\leq C+1,$$

the size of which is (C+1)×(C+1). Let $s_{i}$ be a steady-state probability that the content *k* is staying in state *i* at an arbitrary moment. Then, $s_{k}=\left[s_{1},s_{2},\ldots ,s_{C+1}\right]$ denoting the vector consisting of steady-state probabilities for all states for the content *k* can be obtained by solving the following equation [24]:

(14) $$\begin{array}{cc} & s_{k}\times P_{k}=s_{k}\\ & \mathrm{subject}\;\mathrm{to}\sum_{i=1}^{C+1}s_{i}=1. \end{array}$$

The solution of (14) can be obtained by several approaches and we, in this paper, use a pseudo inverse-based approach [24]. The equation in (14) can be rewritten as

(15) $$\begin{array}{cc} & s_{k}\times \left[P_{k}-I\right]=0\\ & \mathrm{subject}\;\mathrm{to}\sum_{i=1}^{C+1}s_{i}=1, \end{array}$$

where I and 0 denote an identity matrix with the same size as $P_{k}$ and a row vector with (C+1) zeros, respectively. The constraint in (15) can be incorporated into the equation as

(16) $$s_{k}\times \left[(P_{k}-I)|1\right]=\left[0|1\right],$$

by appending 1, which is a column vector with (C+1) ones, to the right of the matrix $\left(P_{i}-I\right)$ and 1 to the right of the vector 0, respectively. Let $\left[(P_{k}-I)|1\right]=Q$ and $\left[0|1\right]=b$, then (16) can be rewritten as $s_{k}Q=b$ and $s_{k}$ can be obtained as

(17) $$s_{k}=bQ^{T}{\left(QQ^{T}\right)}^{-1}.$$

When a client wants to play the video content *k* at an arbitrary moment, the probability that the content is stored in the cache is calculated as

(18) $$c_{k}=\sum_{i=1}^{C}s_{i},$$

while the probability that the content *k* is not stored in the cache at the arbitrary moment is given as $s_{C+1}$ that can be calculated by $s_{C+1}=1-c_{k}$.

**Figure 3:**
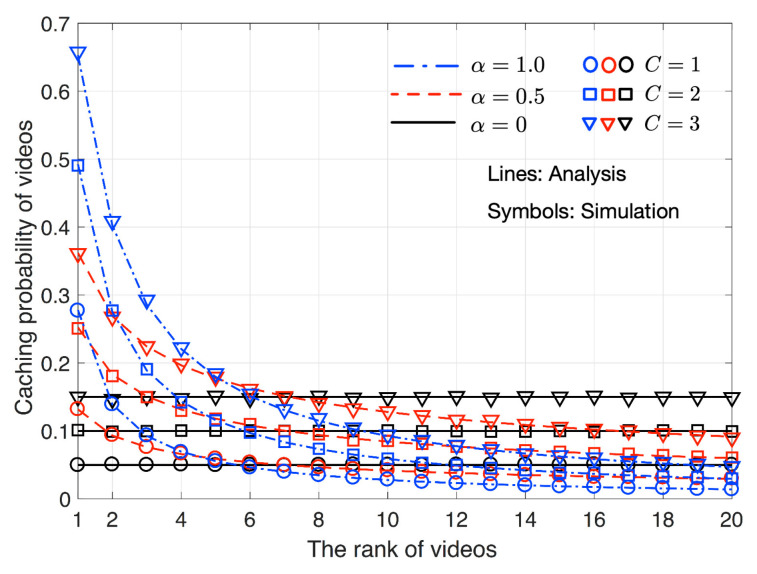
Probability that each content is stored in the cache according to its rank. C∈{1,2,3}, V=10, and α∈{0,1,1.5}.

Figure 3 shows probabilities $c_{k}$’s for various *k* values, C∈{1,2,3}, V=10, and α∈{0,1,1.5}. For C=1, $c_{k}$’s are the same as normalized popularity values given by Zipf distribution because the current content stored in a cache is the content that a client requested according to Zipf distribution. As *C* increases, all $c_{k}$’s increase regardless of the ranks of contents, *k*. As explained in (3), α=0 makes all contents have the same popularity and $c_{k}$’s are thus all the same for all *k*’s. As α increases, $c_{k}$’s of upper rank contents increases while $c_{k}$’s of lower rank contents decrease.

## 3. Proposed Streaming Scheme Using XOR-Based Coding

**Figure 4:**
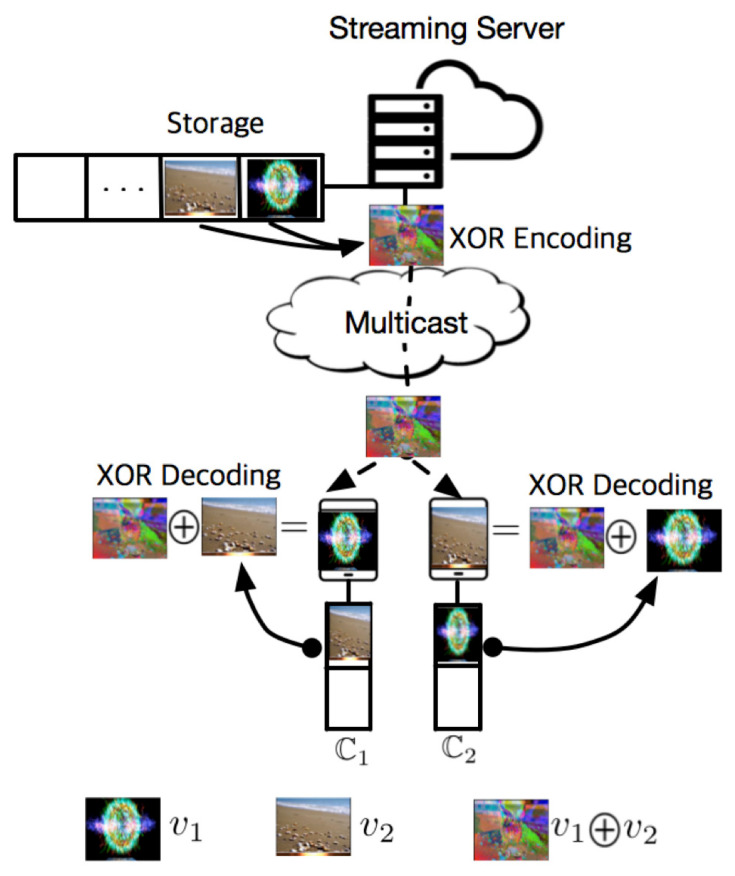
A conceptual illustration of video streaming using XOR-based coding (XC).

The XOR operation has the zero-identity, by which A⊕0=0⊕A=A is satisfied for a given bit stream A, where 0 is the bit stream consisting of 0 and it has the same length as A. It also has the property of self-inverse satisfying A⊕A=0. In addition, XOR is not only commutative, but also associative. Figure 4 illustrates a simplified concept of XC which is a video streaming scheme using XOR coding based on the four aforementioned properties. Client 1 wants to stream $v_{1}$ with $v_{2}$ cached in $C_{1}$, and client 2 wants to stream $v_{2}$ with $v_{1}$ cached in $C_{2}$. Contrary to a conventional scheme where a streaming server transmits $v_{1}$ and $v_{2}$ by UC using two separate connections, XC generates the coded bit stream $v_{1}⊕v_{2}$ and transmits it by using a single connection. Then, client 1 and 2 reconstruct $v_{1}$ and $v_{2}$ from $v_{1}⊕v_{2}$ by using their cached data as

(19) $$\begin{array}{cc} \;\;\;\;\;\left(\;v_{1}\;⊕\;v_{2}\;\right)\;⊕\;v_{2} & \;=\;v_{1}\;⊕\;\left(v_{2}\;⊕\;v_{2}\right)\;=\;v_{1}\;⊕\;0\;=\;v_{1}\\ \;\;\;\;\;\left(\;v_{1}\;⊕\;v_{2}\right)\;⊕\;v_{1} & \;=\;\left(\;v_{2}\;⊕\;v_{1}\right)\;⊕\;v_{1}\;=\;v_{2}\;⊕\;\left(v_{1}\;⊕\;v_{1}\right)\;=\;v_{2}\;⊕\;0\;=\;v_{2}, \end{array}$$

respectively. In this section, we propose a new transmission algorithm for supporting XC while guaranteeing a backward compatibility with conventional streaming schemes such as LC, UC, MC, and BC, that have been widely used to transmit video contents.

### 3.1. A Case Study with Two Clients (N=2)

To clearly explain the proposed transmission scheme, we first consider a special case with two clients, i.e., N=2, and the proposed transmission scheme will be then extended to the general case to support multiple clients, i.e., N≥2, in the following subsection. Suppose that clients 1 and 2 want to play $v_{1}=i$ and $v_{2}=j$, respectively. Each client first checks if the content to play is stored in its cache before requesting the content to a streaming server. If the content is stored in its cache, the client plays the content directly from the cache by LC without an outbound connection. Otherwise, the client transmits a request message including the information of the content to request and the contents of its own cache to a streaming server. Let $\rho_{i,j}^{n}$ be the probability that the number of connections required to transmit *i* and *j* is equal to *n* for given $v_{1}=i$ and $v_{2}=j$, where n∈{0,1,2} and 1≤i,j≤V. For the case of $v_{1}\in C_{1}$ and $v_{2}\in C_{2}$, no connection is required to transmit $v_{1}$ and $v_{2}$. Thus, $\rho_{i,j}^{0}$ can be defined as

(20) $$\rho_{i,j}^{0}=\mathrm{Pr}\left[\left(i\in C_{1}\right)\cap \left(j\in C_{2}\right)\right],$$

and the average total probability that the number of required connections is 0 can be calculated as

(21) $$\mathrm{Pr}\left[n=0\right]=\sum_{i=1}^{V}\sum_{j=1}^{V}f_{i}f_{j}\rho_{i,j}^{0}=\sum_{i=1}^{V}\sum_{j=1}^{V}f_{i}f_{j}c_{i}c_{j},$$

by averaging $\rho_{i,j}^{0}$ over all combinations of *i* and *j*.

For given *i* and *j*, the probability $\rho_{i,j}^{1}$ is defined as

(22a) $$\begin{array}{cc} \rho_{i,j}^{1} & =\mathrm{Pr}[[\left(i\notin C_{1}\right)\cap \left(j\in C_{2}\right)]\cup \left[\left(i\in C_{1}\right)\cap \left(j\notin C_{2}\right)\right] \end{array}$$

(22b) $$\begin{array}{cc} \; & \cup [\left(\;i\;\notin \;C_{1}\right)\;\cap \;\left(\;j\;\notin \;C_{2}\right)\;\cap \;\left(i=j\right)] \end{array}$$

(22c) $$\begin{array}{cc} \; & \cup [\left(\;i\;\notin \;C_{1}\right)\;\cap \;\left(\;j\;\notin \;C_{2}\right)\;\cap \;\left(i\neq j\right)\;\cap \;\left(i\;\in \;C_{2}\right)\;\cap \;\left(j\;\;\in \;C_{1}\;\right)]\;], \end{array}$$

where Equation (22a) denote the probability that only one client is supported by UC because only one of $v_{1}$ and $v_{2}$ is in their own cache. Equation (22b) is the probability that clients are supported by MC because they request the same content, i.e., $v_{1}=v_{2}$, which is not in the clients’ caches. Equation (22c) is the probability that clients are supported by XC because each client does not have the own request content, i.e., $v_{1}\notin C_{1}$ and $v_{2}\notin C_{2}$, but has the content requested by the other client in cache, $v_{1}\in C_{2}$ and $v_{2}\in C_{1}$. In this case, clients 1 and 2 can reconstruct $v_{i}$ and $v_{2}$ from $v_{1}⊕v_{2}$, respectively. Then, the average total probability that the number of required connections is 1 can be calculated as

$$\begin{array}{cc} \;\;\;\;\;\mathrm{Pr}[n=1] & =\sum_{i=1}^{V}\sum_{j=1}^{V}f_{i}f_{j}\rho_{i,j}^{1} \end{array}$$

(23a) $$\begin{array}{cc} \; & =\sum_{i=1}^{V}\sum_{j=1}^{V}f_{i}f_{j}\left[\left(1-c_{i}\right)c_{j}+c_{i}\left(1-c_{j}\right)\right] \end{array}$$

(23b) $$\begin{array}{cc} \; & +\sum_{i=1}^{V}\sum_{j=1,j=i}^{V}f_{i}f_{j}\left[\left(1-c_{i}\right)\left(1-c_{j}\right)\right] \end{array}$$

(23c) $$\begin{array}{cc} \; & +\sum_{i=1}^{V}\sum_{j=1,j\neq i}^{V}f_{i}f_{j}\left[\left(1-c_{i}\right)\left(1-c_{j}\right)c_{j}c_{i}\right]\\ \; & =\sum_{i=1}^{V}\sum_{j=1}^{V}f_{i}f_{j}\left[c_{i}+c_{j}-c_{i}c_{j}\right]\\ \; & +\sum_{i=1}^{V}f_{i}^{2}\left[{\left(1-c_{i}\right)}^{2}\right]\\ \; & +\sum_{i=1}^{V}\sum_{j=1,j\neq i}^{V}f_{i}f_{j}\left[\left(1-c_{i}\right)\left(1-c_{j}\right)c_{j}c_{i}\right], \end{array}$$

by averaging $\rho_{i,j}^{1}$ over all combinations of *i* and *j*.

Finally, for given *i* and *j*, $\rho_{i,j}^{2}$ is defined as

(24) $$\rho_{i,j}^{2}\;\;=\;\;\mathrm{Pr}\left[\left(i\notin C_{1}\right)\cap \left(j\notin C_{2}\right)\cap {\left[\left(i\in C_{2}\right)\cap \left(j\in C_{1}\right)\right]}^{∁}\right],$$

where $A^{∁}$ denotes the complement of a set *A*. Then, the average total probability that the number of required connections is 2 can be calculated as

(25) $$\begin{array}{cc} \;\;\;\;\;\mathrm{Pr}[n=2]\; & \;=\;\sum_{i=1}^{V}\sum_{j=1}^{V}f_{i}f_{j}\rho_{i,j}^{2}\\ \; & =\sum_{i=1}^{V}\sum_{j=1}^{V}f_{i}f_{j}\left[\left(1-c_{i}\right)\left(1-c_{j}\right)\left(1-c_{j}c_{i}\right)\right], \end{array}$$

by averaging $\rho_{i,j}^{2}$ over all combinations of *i* and *j*. In this case, clients are supported by UC through two independent connections, respectively.

### 3.2. A Transmission Algorithm Extended for Multiple Clients (N≥2)

In this subsection, we propose a transmission algorithm by extending the transmission for two clients described in the previous subsection in order to support XC for N(N≥2) clients, while guaranteeing a backward compatibility with conventional streaming schemes such as LC, UC, and MC. Our algorithm consists of two phases. In the first phase, a client n(1≤n≤N) is categorized into LC or MC based on the requesting content, $v_{n}$, and cache status, $C_{n}$, or proceeds to the second phase. The clients satisfying $v_{n}\in C_{n}$ will be served by LC and become the elements of $U_{L}$, which is a set of clients served by LC. The remaining clients with $v_{n}\notin C_{n}$, given by $T=\left\{1,\ldots ,N\right\}\backslash U_{L}$, transmit a request message to a streaming server including a content to play and cache information. If two or more clients request the same video content, they are all categorized into an MC group. The MC group becomes an element of $G_{M}$, which is a set consisting of MC groups, and the cardinality of $G_{M}$ is $0\leq |G_{M}|\leq \left\lfloor \frac{\left|T\right|}{2}\right\rfloor$ because an MC group consists of at least two clients. $U_{M}$ is composed of all clients included in $G_{M}$.

In the second phase, the clients who are not included in either $U_{L}$ or $U_{M}$, given by $T\;=\;\left\{1,\ldots ,N\right\}\backslash U_{L}\backslash U_{M}$, are categorized into XC or UC groups. A client *n* in T is first checked to see if it can be served by XC. For an existing XC group *S*, the client *n* can join the existing XC group *S* only if the following conditions are satisfied:

(26) $$\begin{array}{cc} C_{n} & ∋v_{m}\;\forall m\in S,\\ v_{n} & \in C_{m}\;\forall m\in S. \end{array}$$

If there is no existing XC group or the client *n* fails to join any existing XC group, the client *n* attempts to create a new XC group with another client *m* who is included in T and satisfies

(27) $$\begin{array}{cc} v_{m} & \in C_{n},\\ C_{m} & ∋v_{n}. \end{array}$$

This process is repeated for all clients in T.

For the case that the client *n* succeeds to join the existing XC group or to create the new XC group, the client *n* becomes an element of $U_{X}$, which is a set consisting the clients in XC groups. Finally, the clients who are not included in either $U_{L}$, $U_{M}$, or $U_{X}$ are included into $U_{U}$ and will be served by UC through separate connections. The proposed video streaming scheme using XC is explained in detail in the Algorithms 1 and 2.

**Algorithm 1** Proposed Video Streaming:**Phase 1.** LC and MC1: **Initialization:**2:   $U_{L}=\emptyset ,\;U_{M}=\emptyset$          ▹ Sets of clients3:   $G_{M}=\emptyset$          ▹ Set of MC groups4: **Obtain a set of candidate clients for LC:**5:   T={1,…,N}6: **for**
n∈T
**do**7:     **if**
$v_{n}\in C_{n}$
**then**8:       $U_{L}=U_{L}\cup \left\{n\right\}$9:     **end if**10. **end for**11: **Obtain a set of candidate clients for MC:**12:   $T=\left\{1,\ldots ,N\right\}\backslash U_{L}$13: **while**
|T|>0
**do**14:     u=T[0]15:     S=∅16:     **for**
m∈T
**do**17:       **if**
$v_{u}==v_{m}$
**then**18:         S=S∪{m}19:       **end if**20:     **end for**21:     **if**
|S|>=2
**then**22:       $G_{M}=G_{M}\cup \left\{S\right\}$23:       $U_{M}=U_{M}\cup S$24:       T=T\S25:     **end if**26: **end while**

**Algorithm 2** Proposed Video Streaming:**Phase 2.** XC and UC27: **Initialization:**28:   $U_{X}=\emptyset$          ▹ Set of clients29:   $G_{X}=\emptyset$          ▹ Set of XC groups30: **Obtain a set of candidate clients for XC:**31:   $T=\left\{1,\ldots ,N\right\}\backslash U_{L}\backslash U_{M}$32: cnt=033: **while**
cnt<|T| and |T|>0
**do**34:     $cnt^{++}$35:     u=T[0]          ▹ A client to check for XC.36:     **if**
$|G_{X}|>0$**then**          ▹ XOR group already exists.37:       **for**
$S\in G_{X}$**do**        ▹*S* is a set.38:         j=True39:         **for**
s∈S
**do**40:           j *= $\left(v_{u}\in C_{s}\right)*\left(v_{s}\in C_{u}\right)$41:         **end for**42：        **if**
j==True
**then**43：           S=S∪{u}      ▹ Client *u* is added into *S*.44：           $U_{X}=U_{X}\cup \left\{u\right\}$45：           T=T\{u}46：          **break**47：        **end if**48：      **end for**49：    **end if**50:     **if** ($|G_{X}\left|==0\right)$ or (j==False)
**then**51:       **for**
s∈T\{u}
**do**52:         **if**
$\left(v_{u}\in C_{s}\right)*\left(v_{s}\in C_{u}\right)$
**then**53:           $G_{X}\;\;=\;\;G_{X}\cup \left\{\{u,s\}\right\}$     ▹ A new group of XC54:           T=T\{u,s}55:           $U_{X}=U_{X}\cup \left\{u,s\right\}$56:           **break**57:         **end if**58:       **end for**59:     **end if**60: **end while**61: $U_{U}=\left\{1,\ldots ,N\right\}\backslash U_{L}\backslash U_{M}\backslash U_{X}$

## 4. Numerical Results

In this section, the performance of video streaming scheme using XC is analyzed in terms of average connections required to serve each client’s video play, and compared with that of a conventional scheme without XC. Let κ denote the number of the required connections of the video streaming schemes. For the proposed scheme using XC, κ can be calculated as

(28) $$\kappa_{\mathrm{prop}}=|G_{M}\left|+\right|G_{X}\left|+\right|U_{U}|,\;\;0\leq \kappa_{\mathrm{prop}}\leq N.$$

For the conventional scheme without XC, we need to allocate a dedicated connection to every client in $U_{X}$. Thus, κ for the conventional scheme can be calculated as

(29) $$\kappa_{\mathrm{conv}}=|G_{M}\left|+\right|U_{X}\left|+\right|U_{U}|,\;\;0\leq \kappa_{\mathrm{conv}}\leq N.$$

Please note that $\kappa_{\mathrm{prop}}\leq \kappa_{\mathrm{conv}}$ is always satisfied because $|G_{X}\left|\leq \right|U_{X}|$ and $|G_{X}\left|=\right|U_{X}|$ is only valid when $|G_{X}\left|=\right|U_{X}|=0$. We define the average normalized load as the average number of required connections per each client’s video view, which is denoted by η and can be calculated for the proposed and conventional schemes, respectively, as

(30) $$\begin{array}{cc} \eta_{\mathrm{prop}} & =\sum_{n=0}^{N}\frac{n}{N}\times \mathrm{Pr}\left[\kappa_{\mathrm{prop}}=n\right],\\ \eta_{\mathrm{conv}} & =\sum_{n=0}^{N}\frac{n}{N}\times \mathrm{Pr}\left[\kappa_{\mathrm{conv}}=n\right]. \end{array}$$

All numerical results were obtained through mathematical analysis given in Section 3.1 and verified by Monte-Carlo simulations using a Python-based simulator. In the figures, the lines and markers denote numerical results from mathematical analysis and Monte-Carlo simulations, respectively. In simulations, the number of iterations for averaging the effect of many random variables is 100,000.

**Figure 5:**
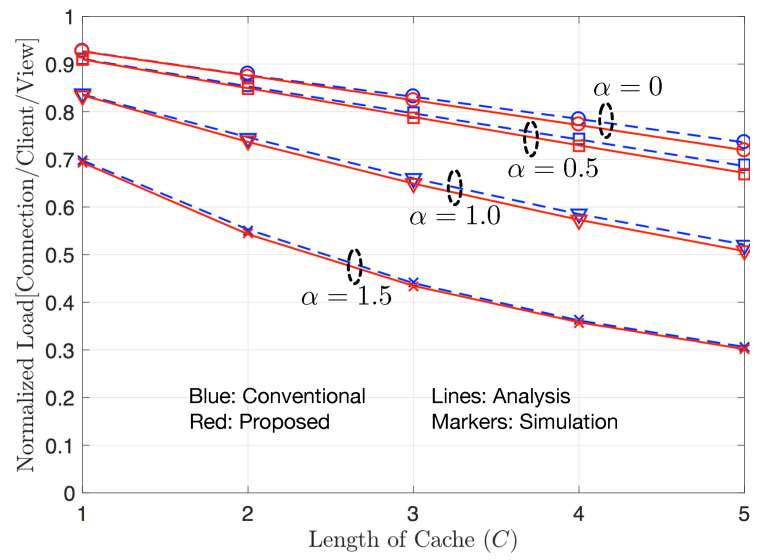
Average normalized load for various values of *C*. *N*=2, V=20, and α∈{0,0.5,1.0,1.5}.

Figure 5 shows the average normalized load η of the proposed streaming scheme using XC and the conventional scheme without XC for various values of cache length. The numbers of clients and video contents are given by N=2 and V=20, respectively, and the parameter α of Zipf distribution for contents’ normalized popularity is 0, 0.5, 1.0, or 1.5. The figure shows that our numerical results obtained by mathematical analysis are consistent with those from simulations. The proposed and conventional schemes can both reduce the average load as the parameter α of Zipf distribution increases. As α increases, the deviation of popularity among contents is expanded and thus every client is more likely to play the popular contents, which indicates that the probabilities that the popular contents are cached increase while the probabilities that less popular contents are cached decrease. As a result, an increasing α can reduce the average normalized load because the hit probability, which is the probability that the contents to play are cached in their own caches, increases. As the length of cache, *C*, increases, the probabilities that contents are cached increase regardless of popularity. Thus, for given α, an increasing *C* also reduces the average normalized load. To be more specific, when α=1.5 and *C* increases from 1 to 5, the average normalized load decreases from 0.7 to 0.3 for both proposed and conventional schemes. In this figure, although the proposed scheme using XC outperforms the conventional scheme without XC, the gap of the performance is marginal, because when the number of clients is small (N=2), the frequency of using XC decreases and hence, the gain achieved by XC is limited.

**Figure 6:**
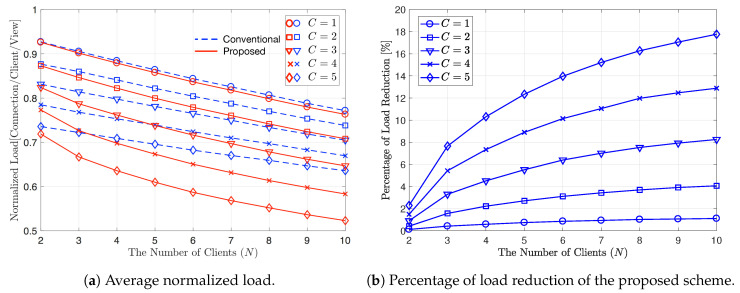
Average normalized load and percentage of load reduction of the proposed scheme for varying *N*. V=20, C∈{1,2,3,4,5}, and α=0.

In Figure 6, we compare the performance of the proposed scheme to the conventional scheme according to the number of clients, *N*. Figure 6a shows the average normalized load of the proposed and conventional schemes by varying *N* from 2 to 10 when V=20, C∈{1,2,3,4,5} and α=0. Here, α=0 means that Zipf distribution become Uniform distribution and all video contents thus have the same popularity. As *N* increases, the frequency of using MC for the conventional scheme and the frequency of using MC or XC for the proposed scheme both increase. Thus, the average normalized load decreases with an increasing *N* for both the proposed and conventional schemes, as predicted in Figure 5. In addition, the average load also decreases with an increasing *C* because the frequency of using LC for the conventional scheme and the frequency of using LC or XC for the proposed scheme both increase as *C* increases. Figure 6b shows how much the proposed scheme can reduce the average normalized load by using XC, compared to the conventional scheme. The percentage of average load reduction by the proposed scheme is calculated as $\zeta =\frac{\eta_{\mathrm{conv}}-\eta_{\mathrm{prov}}}{\eta_{\mathrm{conv}}}\times 100\left[\%\right]$. In this figure, we can observe that for all *N* and *C* values, the proposed scheme outperforms the conventional scheme due to the gain obtained from XC, and thus ζ>0,∀N,C. The frequency of using XC in the proposed scheme increases as *N* or *C* increases and thus, ζ increases with increasing *N* or *C*. As a specific example with C=5 and N=10, the proposed scheme can reduce the average load about 18%, compared to the conventional scheme.

**Figure 7:**
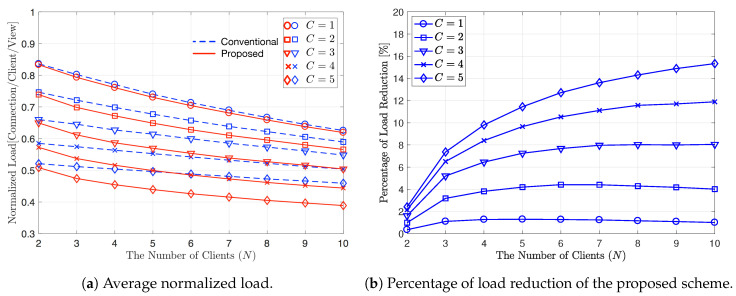
Average normalized load and percentage of load reduction of the proposed scheme for varying *N*. V=20, C∈{1,2,3,4,5}, and α=1.0.

In Figure 7, according to *N*, we plot the average normalized load in Figure 7a and the percentage of average load reduction, ζ, in Figure 7b, respectively, when V=20 and C∈{1,2,3,4,5}. In this figure, α is set to 1.0 to let all videos have different popularity from each other. For the case of α=1.0, the frequency of viewing top three out of 20 videos accounts for about 50%. The average normalized load is reduced more for both proposed and conventional schemes as α increases from 0 to 1.0, as shown in Figure 6a and Figure 7a. As α increases, the deviation in popularity among video contents increases, which can yield the same effect as increasing *N* or *C*. By comparing Figure 6a and Figure 7a, we can observe that as α increases from 0 to 1.0, the average load of the proposed scheme decreases from 0.523 to 0.39 when C=5 and N=10. In terms of the percentage of average load reduction, by comparing Figure 6b and Figure 7b, we can observe that ζ is determined by *C* and *N* as well as α. For the case of N=10, when *C* is relatively large, e.g., C=5, ζ decreases from 17.8% to 15.3% as α increases from 0 to 1.0. In this case, however, when C=1,2, or 3, ζ with α=1.0 is greater than ζ with α=0 for most *N* values. For example, when C=3 and N=5, ζ increases from 5.5% to 7.3% as α increases from 0 to 1.0. In the proposed scheme, LC and MC are given a higher priority than XC. Therefore, as *C*, *N*, and α all increase, the probability of using LC or MC increases and the gain achieved from XC decreases slightly. Even though we used small values for *N*, *V*, and *C* to simplify computer simulations, the proposed scheme can be applied to real large-scaled video stream systems.

## 5. Conclusions

A streaming server can process requests from multiple clients simultaneously by coding the bit streams requested from the multiple clients with XOR operation and multicasting the coded bit stream to the multiple clients. Each client can recover its content from the coded bit stream with XOR operation using contents stored in its cache, which is based on zero-identity and self-inverse properties of XOR and can reduce the number of transmissions by dealing with the requests simultaneously. Existing studies on the data transmission using XOR-based coding mainly focused on theoretical analysis or optimal coding in limited environments, and most of them were thus less feasible. In this paper, we investigated how the data transmission using XOR-based coding could be applied to video streaming systems without interfering existing video streaming methods such as UC, MC, and BC. We first modeled the behavior of video contents in clients’ caches as a Markov chain, and derived the caching probability that a video content has been already cached in the client’s cache when a client wants to play the content at an arbitrary moment. We also proposed a new video streaming scheme for supporting XC while guaranteeing a backward compatibility with the conventional video streaming methods. With a simplified case of two clients, the basic concept of the proposed scheme was described, and the performance of the proposed scheme mathematically analyzed in terms of average normalized load. All analytical results were verified by computer simulations. Then, the proposed scheme was extended to a complete algorithm that can support multiple clients more than two. Our numerical results showed that the proposed scheme using XC outperforms the conventional scheme without XC in all situations and the performance enhancement of the proposed scheme becomes more noticeable as the number of clients or the length of cache increase. For a specific situation with C=5 and N=10, we showed the proposed scheme can reduce the average load about 18%, compared to the conventional scheme.
